# Pre-Existing Hypoxia Is Associated with Greater EEG Suppression and Early Onset of Evolving Seizure Activity during Brief Repeated Asphyxia in Near-Term Fetal Sheep

**DOI:** 10.1371/journal.pone.0073895

**Published:** 2013-08-21

**Authors:** Guido Wassink, Laura Bennet, Joanne O. Davidson, Jenny A. Westgate, Alistair J. Gunn

**Affiliations:** 1 Department of Physiology, University of Auckland, Auckland, New Zealand; 2 Department of Obstetrics and Gynaecology, Auckland, New Zealand; Université de Montréal, Canada

## Abstract

Spontaneous antenatal hypoxia is associated with high risk of adverse outcomes, however, there is little information on neural adaptation to labor-like insults. Chronically instrumented near-term sheep fetuses (125 ± 3 days, mean ± SEM) with baseline PaO_2_ < 17 mmHg (hypoxic group: n = 8) or > 17 mmHg (normoxic group: n = 8) received 1-minute umbilical cord occlusions repeated every 5 minutes for a total of 4 hours, or until mean arterial blood pressure (MAP) fell below 20 mmHg for two successive occlusions. 5/8 fetuses with pre-existing hypoxia were unable to complete the full series of occlusions (vs. 0/8 normoxic fetuses). Pre-existing hypoxia was associated with progressive metabolic acidosis (nadir: pH 7.08 ± 0.04 vs. 7.33 ± 0.02, p<0.01), hypotension during occlusions (nadir: 24.7 ± 1.8 vs. 51.4 ± 3.2 mmHg, p<0.01), lower carotid blood flow during occlusions (23.6 ± 6.1 vs. 63.0 ± 4.8 mL/min, p<0.01), greater suppression of EEG activity during, between, and after occlusions (p<0.01) and slower resolution of cortical impedance, an index of cytotoxic edema. No normoxic fetuses, but 4/8 hypoxic fetuses developed seizures 148 ± 45 minutes after the start of occlusions, with a seizure burden of 26 ± 6 sec during the inter-occlusion period, and 15.1 ± 3.4 min/h in the first 6 hours of recovery. In conclusion, in fetuses with pre-existing hypoxia, repeated brief asphyxia at a rate consistent with early labor is associated with hypotension, cephalic hypoperfusion, greater EEG suppression, inter-occlusion seizures, and more sustained cytotoxic edema, consistent with early onset of neural injury.

## Introduction

Chronic antenatal hypoxia due to placental dysfunction or multiple pregnancies is commonly associated with growth retardation and a higher risk of stillbirth [1], and long-term abnormal neurodevelopmental outcome [2]. It is unclear to what extent such pre-existing hypoxia contributes to greater risk of acute perinatal brain injury. We have previously shown that in fetal sheep with pre-existing stable hypoxia even brief umbilical cord occlusions, repeated at a rate consistent with early labor, are associated with severe metabolic acidosis and hypotension [3]. These observations clearly indicate that chronic hypoxia is associated with reduced cardiac tolerance to labor-like asphyxia, but the effects on cerebral perfusion and neural adaptation are unclear.

The rapid initial suppression of EEG activity during severe hypoxia and asphyxia, before the onset of neural depolarization [4], reflects active suppression of brain metabolism [5–8] that helps delay the onset of cytotoxic edema and reduces neuronal injury [9]. This neuroprotective response has also been described during repeated umbilical cord occlusions in healthy near-term fetal sheep, with rapid and reversible suppression of EEG activity approximately 90 seconds after the onset of each occlusion, with superior sagittal sinus blood flow and arterial blood pressure values that were maintained at or above baseline values [10,11]. In contrast, Frash et al. observed EEG suppression only when one-minute occlusions were repeated every 2 minutes, and coincided with worsening acidemia and hypotension [12]. In addition, De Haan et al. reported that suppression of EEG activity was both faster and more profound during 2-minute occlusions repeated every 5 minutes compared to 1-minute occlusions repeated every 2.5 minutes, and associated with greater frequency of epileptiform and spike activity between occlusions [13]. These findings strongly suggest that the extent of neural adaptation and aberrant EEG activity during repeated brief asphyxia relates to the development of systemic compromise, and thus may be linked directly to pre-existing fetal condition.

Therefore, in the present study we examined the hypothesis that in near-term fetal sheep with pre-existing stable hypoxia, one minute umbilical cord occlusions repeated every five minutes, a rate consistent with early labor, is associated with cerebral hypoperfusion and cytotoxic edema, greater EEG suppression, and development of abnormal EEG activity.

## Materials and Methods

### Surgical preparation and post-operative care

All animal procedures were approved by the Animal Ethics Committee of the University of Auckland, New Zealand. 16 time-mated Romney/Suffolk cross-breed fetal sheep were instrumented between 119 and 122 days gestation (term = 147 days) using sterile technique as described earlier [14]. We have previously reported changes in fetal heart rate (FHR), mean arterial blood pressure (MAP), T/QRS ratio, and blood composition for a subset of animals from this experimental group [3]. Food, but not water was withdrawn 18 h before surgery. Ewes were given 5 mL of Streptopen (procaine penicillin (250,000 IU/mL) and dihydrostreptomycin (250 mg/mL, Stockguard Labs Ltd., Hamilton, New Zealand)) intramuscularly for prophylaxis 30 min prior to the start of surgery. Anesthesia was induced by intravenous (i.v.) injection of Alfaxan (alphaxalone, 3 mg/kg, Jurox, Rutherford, NSW, Australia), and general anesthesia maintained using 2-3% isoflurane in O_2_. The depth of anesthesia and maternal respiration were constantly monitored by trained anesthetic staff, and the ewes received a constant infusion of isotonic saline to maintain maternal fluid balance.

Following a maternal midline abdominal incision and exteriorization of the fetus, polyvinyl catheters were placed in the left and right brachial artery and right vein to measure arterial and venous blood pressure and to obtain fetal arterial blood samples. An amniotic catheter was secured to the fetal shoulder. Electrocardiogram (ECG) electrodes (AS633-3SSF, Cooner Wire Co., Chatsworth, CA, USA) were placed subcutaneously over the right shoulder and chest at apex level and sewn across the chest to record the fetal ECG. An ultrasound blood flow probe (3S-mm, Transonic Systems Inc., Ithaca, NY, USA) was placed around the left carotid artery to measure carotid blood flow (CaBF) as an index of global cephalic blood flow [8,15,16].

Two pairs of EEG electrodes (AS633-7SSF, Cooner Wire Co.) were placed on the dura over the parasagittal parietal cortex (5 mm and 15 mm anterior to bregma and 10 mm lateral) and secured with cyanoacrylate glue. To measure cortical impedance, a third pair of electrodes (AS633-3SSF, Cooner Wire Co.) was placed over the dura 5 mm lateral to the EEG electrodes, and a reference electrode was sewn over the occiput. An inflatable silicone occluder was loosely fitted around the umbilical cord of all fetuses (In Vivo Metric, Healdsburg, CA, USA). The uterus was then closed and Gentamicin (80 mg/2mL, Rousell, Auckland, New Zealand) was administered into the amniotic sac. Any amniotic fluid lost during surgery was replaced using isotonic saline warmed to 37°C. The maternal abdominal wall and skin was repaired and the skin incision infiltrated with 10 mL of local acting analgesic Marcaine (0.5% bupivacaine plus adrenaline, AstraZeneca Ltd., Auckland, New Zealand). All leads were then exteriorized through the maternal flank and a maternal long saphenous vein was catheterized to provide access for post-operative care and euthanasia. During the surgical procedures, care was taken to avoid damage to the placental cotyledons, to make small incisions that followed the line of uterine blood flow, to withdraw fetuses as little as possible and to replace them *in situ* in their natural lie, to avoid any twisting of the fetal umbilical cord. Surgery was completed in 2.5 hours or less in all cases.

Post-operatively all ewes were housed in individual metabolic wooden cages with access to water and concentrate pellet feed (Country Harvest Stockfeed, Cambridge, New Zealand) *ad libitum*. The animal housing facility was climate-controlled (temperature; 16 ± 1°C, humidity; 50-60%), and operated on a 12 hour light/dark cycle at all times. During the post-operative recovery period all ewes were given i.v. antibiotics, including gentamicin (80 mg/2 mL for 2 days, Rousell) and benzylpenicillin sodium (600 mg for 4 days, Novartis Ltd., Auckland, New Zealand). Fetal catheters were maintained patent by continuous infusion of heparinized saline (20 U/mL^-1^ at 0.2 mL/h), and the maternal catheter maintained by daily flushing with heparinized saline. All fetuses were monitored daily after surgery. Only fetuses with normal sleep state cycling and no seizures before the experiment were studied. Experiments were initiated 3 to 6 days after surgery (125 ± 1 days gestation). Three days after the occlusion series, ewes and fetuses were killed with an intravenous overdose of pentobarbitone sodium (9 g) to the ewe (Pentobarb 300; Chemstock International, Christchurch, New Zealand). Fetuses were removed by hysterectomy and weighed. Histology is unavailable in the present study due to a high mortality rate in the hypoxic group.

### Data acquisition

Fetal MAP, corrected for maternal movement by subtraction of amniotic pressure (Novatrans II, MX860; Medex Inc., Hilliard, OH, USA), CaBF, ECG, EEG, and impedance were recorded continuously, from 24 h before the experiment until fetal death. The MAP signal was collected at 64 Hz and low-pass filtered at 30 Hz. Carotid blood flow was measured as an index of changes in global cephalic blood flow [17]. The raw ECG was analog filtered between 0.05 and 80 Hz and digitized at 512 Hz. The EEG signal was processed with a first-order high-pass filter at 1.6 Hz and a 6th order Butterworth low-pass filter with a cut-off frequency at 50 Hz, and then digitally stored at a sampling rate of 64 Hz. EEG intensity (power) was derived from the power spectrum signal between 1 and 20 Hz, and log transformed (decibels (dB), 20 x log (intensity)), as this transformation gives a better approximation of normal distribution [18]. All experimental data were collected using customized acquisition software (Labview for Windows, National Instruments Ltd., Austin, Tx, USA) for off-line analysis.

### Experimental design

Arterial blood gases were measured daily after fetal instrumentation. Fetuses from singleton (n = 1), twin (n = 5) or triplet (n = 2) pregnancies with stable PaO_2_ less than 17 mmHg for at least 2 days were assigned to the hypoxic group (n = 8). The normoxic group (n = 8) consisted of healthy twin (n = 4) and singleton pregnancies (n = 4) with an arterial PaO_2_ greater than 17 mmHg. Repeated umbilical cord occlusion was achieved by inflating the umbilical occluder with sterile saline for 60 seconds followed by a 4 minute recovery period (inter-occlusion period), after which the next occlusion was performed. This procedure was repeated for up to four hours (equal to 49 occlusions), or until the MAP had fallen below 20 mmHg during two successive occlusions. Fetal blood gas analysis (Ciba-Corning diagnostics 845 blood gas analyzer, MA, USA) and measurements of glucose and lactate levels (YSI model 2300, Yellow Springs, OH, USA) were performed before (baseline) and after the first occlusion, after every 12^th^ occlusion, following the last occlusion, 30 minutes, one, four and 24 hours after occlusions.

### Data analysis and statistical procedures

One second averaged MAP and CaBF data were used to determine the maximum value at the beginning of each occlusion, the minimum value at the end of each occlusion, and the mean inter-occlusion MAP and CaBF, calculated from the 2^nd^ minute until the start of the next occlusion. Cephalic oxygen delivery was calculated as fetal arterial oxygen content (O_2_ct) x CaBF. For impedance, the peak value of each occlusion was determined using one minute averaged data and expressed as percent change from baseline, calculated from the mean 12 hour period before the start of the occlusion series. The impedance of tissue rises concomitantly as cells depolarize and fluid shifts from the extracellular to intracellular space, and thus impedance is a functional measure of cytotoxic edema [13,19]. One second averaged EEG power data (derived from non-overlapping epochs) was normalized by subtraction of the mean 12 hour baseline period, and used to identify the maximum EEG suppression of each occlusion, determined as the nadir that occurred within 90 seconds from the start of occlusion. To reduce variance from suppression or seizures at the end of occlusion, the mean inter-occlusion EEG was calculated from the 3^rd^ minute until the start of the next occlusion. For each parameter, the baseline period was taken as the mean of 6 hours before the first occlusion. Overt electrographic seizures were identified visually and defined as the concurrent appearance of sudden, repetitive, evolving stereotyped waveforms in the EEG signal, lasting more than 10 seconds and >20 µV [20]. Seizure-like events were defined as similar in appearance to stereotypic seizures, but lacking its evolutionary pattern. Seizure burden was calculated as the mean time of seizure activity between each occlusion or per hour of recovery. Two animals in the normoxic group were not included in the EEG power analysis due to excessive artifact on the recordings, while one normoxic animal was not included for analysis of seizures and sleep state cycling. Sleep state cycling was determined to the nearest hour from 1-minute EEG data as a repetitive alternating pattern of high and low-voltage activity, with each phase lasting approximately 20-30 minutes. Time of death was taken for animals that did not recover sleep state cycling.

Because some fetuses in the hypoxic group were unable to complete the full 4 hour period before MAP fell below 20 mmHg during two successive occlusions, the occlusion series are represented as the means of four quarters of the total occlusion period in order to allow comparison with the normoxic group. Each quarter included 9 occlusions per animal; the first quarter begins with the first occlusion and the last quarter ends with the final occlusion for each fetus. The middle two quarters were defined as the median of the respective interval ± 4 occlusions. The effects of hypoxia and occlusions on physiological changes were assessed by analysis of variance (ANOVA, SPSS v12, SPSS Inc., Chicago, IL, USA), with time treated as a repeated measure. The baseline was used as a covariate for MAP and CaBF. When statistical significance was detected, post-hoc comparisons were performed with univariate analysis and Fisher’s Least Significant Difference or Dunnett’s test. Data not normally distributed were compared by non-parametric Mann–Whitney U test. All data are mean ± SEM.

## Results

There was no difference in the 6 hour baseline measurements of CaBF, EEG power, or impedance between groups, but MAP was lower in the hypoxic group (41.4 ± 1.5 vs. 46.4 ± 0.8 mmHg, p<0.05). Gestational age and gender were similar between groups, but fetuses in the hypoxic group were significantly smaller at post-mortem (3273 ± 226 vs. 4040 ± 141 g, p<0.05, Table 1). Five of 8 fetuses in the hypoxic group were discontinued early (at occlusion numbers 34, 39, 44, 44, and 45 respectively), when MAP had fallen below 20 mmHg during two successive occlusions. Five fetuses in the hypoxic group died early in the recovery period because of deteriorating condition (mean survival time; 25.3 ± 5.8 vs. 68.0 ± 1.1 hours after final occlusion, p<0.01).

**Table 1:** Experimental groups.

Group	n	Gestation (days)	Weight (g)	Singleton : Twin : Triplet	Sex (female : male)
N	8	126.4 ± 0.9	4040 ± 141	4 : 4 : 0	5 : 3
H	8	124.0 ± 0.9	3273 ± 226*	1 : 5 : 2	3 : 5

Normoxic (N: n = 8) and Hypoxic (H: n = 8) groups. Data are Mean ± SEM; between-group comparisons by Mann–Whitney U test. ^*^P <0.05, ^†^P <0.01.

### Blood composition analysis

The hypoxic group had stable but significantly lower baseline pH, PaO_2_ and O_2_ct values (p<0.01), and higher PaCO_2_ (p<0.05) than the normoxic group (Table 2). In the normoxic group, brief repeated umbilical cord occlusions were associated with a minor fall in pH and O_2_ct, and a moderate rise in base deficit and lactate, whereas pre-existing hypoxia was associated with severe mixed respiratory and metabolic acidosis that progressively worsened throughout the occlusion series (Table 2), with a greater rise in lactate (p<0.05 vs. normoxic group). All parameters had recovered to their respective baseline levels within 24 hours after the final occlusion.

**Table 2:** Fetal blood composition parameters in the Normoxic (N; n = 8) and Hypoxic (H; n = 8) groups.

		**Baseline**	**Occlusion**					**Recovery**			
			**First**	**12**	**24**	**36**	**Final**	**+30 Min**	**+60 Min**	**+4 Hours**	**+24 Hours**
**pH**	**N**	7.40 ± 0.01	7.37 ± 0.02	7.33 ± 0.02	7.31 ± 0.03	7.32 ± 0.03	7.32 ± 0.03	7.39 ± 0.02	7.40 ± 0.01	7.43 ± 0.01	7.39 ± 0.01
	**H**	7.37±0.01†	7.33 ± 0.02	7.13 ± 0.03†	7.09±0.04†	7.05±0.06†	7.07 ± 0.05†	7.14 ± 0.06†	7.18 ± 0.05†	7.30 ± 0.05†	7.40 ± 0.01
**PaCO_2_**	**N**	45.5 ± 1.0	48.2 ± 2.0	47.4 ± 1.4	46.3 ± 2.4	47.0 ± 2.2	46.3 ± 1.4	43.3 ± 1.0	43.0 ± 1.1	44.9 ± 1.6	46.5 ± 1.1
mmHg	**H**	50.2 ± 1.3*	52.9 ± 2.7	60.0 ± 2.8†	52.3 ± 2.3	55.3 ± 2.9*	54.2 ± 3.4*	47.6 ± 2.9	47.2 ± 2.2	49.6 ± 2.5	50.8 ± 1.6*
**PaO_2_**	**N**	21.4 ± 0.6	19.3 ± 1.5	17.8 ± 1.2	18.0 ± 1.1	16.9 ± 0.9	16.6 ± 0.6	20.3 ± 1.0	19.6 ± 1.0	19.9 ± 0.8	21.2 ± 0.7
mmHg	**H**	11.4 ± 0.9†	11.9 ± 0.7†	14.2 ± 0.8*	15.4 ± 0.7	14.5 ± 0.5*	14.4 ± 1.2	13.9 ± 0.7†	13.6 ± 0.6†	11.6 ± 0.5†	11.1 ± 1.1†
**O_2_ct**	**N**	4.3 ± 0.3	3.4 ± 0.3	3.3 ± 0.3	3.3 ± 0.2	3.0 ± 0.2	3.0 ± 0.2	4.1 ± 0.4	4.0 ± 0.3	3.9 ± 0.3	4.2 ± 0.3
mmol/L	**H**	1.8 ± 0.3†	1.8 ± 0.2†	1.6 ± 0.2†	1.6 ± 0.2†	1.4 ± 0.1†	1.5 ± 0.2†	1.6 ± 0.1†	1.7 ± 0.2†	1.6 ± 0.2†	1.6 ± 0.2†
**DO_2_**	**N**	208 ± 23	166 ± 23	193 ± 8	208 ± 28	179 ± 18	189 ± 24	176 ± 25	191 ± 21	186 ± 12	228 ± 19
µmol/min	**H**	122 ± 24*	71 ± 15*	63 ± 22†	59 ± 23†	41 ± 21†	40 ± 15†	104 ± 21*	98 ± 15†	91 ± 16†	99 ± 6*
**BD**	**N**	-2.9 ± 0.6	-1.5 ± 0.8	2.3 ± 1.1	3.4 ± 1.3	3.1 ± 1.1	2.5 ± 1.4	-1.0 ± 1.0	-1.6 ± 0.8	-4.2 ± 1.0	-2.2 ± 0.4
mmol/L	**H**	-2.4 ± 0.7	-0.4 ± 0.9	9.9 ± 1.2†	13.7 ± 1.6†	15.2 ± 1.9†	14.5 ± 1.7†	12.8 ± 2.0†	10.2 ± 2.0†	2.3 ± 2.5*	-5.2 ± 2.0
**Lactate**	**N**	1.0 ± 0.1	1.6 ± 0.4	3.1 ± 0.8	4.1 ± 1.2	4.5 ± 1.3	5.2 ± 1.6	4.5 ± 1.4	3.6 ± 1.1	1.6 ± 0.4	0.8 ± 0.1
mmol/L	**H**	2.5 ± 0.6	3.5 ± 0.6	8.8 ± 1.4*	10.2 ± 1.6*	10.5 ± 2.2	9.3 ± 2.2	10.6 ± 2.1	10.0 ± 1.7*	5.9 ± 1.6*	1.8 ± 0.4*
**Glucose**	**N**	1.0 ± 0.2	1.0 ± 0.3	1.4 ± 0.2	1.3 ± 0.2	1.4 ± 0.2	1.4 ± 0.3	1.4 ± 0.2	1.3 ± 0.2	1.2 ± 0.2	1.1 ± 0.2
mmol/L	**H**	0.7 ± 0.1	0.9 ± 0.2	1.3 ± 0.2	1.2 ± 0.1	1.1 ± 0.1	1.1 ± 0.2	1.0 ± 0.2	1.0 ± 0.2	1.0 ± 0.2	0.8 ± 0.1

Data are Mean ± SEM; between-group comparisons by Mann–Whitney U test. Fetal blood samples were taken before the experiment, every twelfth occlusion, and at 30 minutes, 60 minutes, 4 hours, and 24 hours after the occlusion series. PaCO_2_, fetal arterial pressure of carbon dioxide; PaO_2_, fetal arterial pressure of oxygen; O_2_ct, fetal arterial oxygen content; DO_2_, cephalic oxygen delivery; BD, base deficit; Min, minutes. *P<0.05, ^†^P<0.01.

MAP and CaBF during brief repeated occlusions

**Figure 1:**
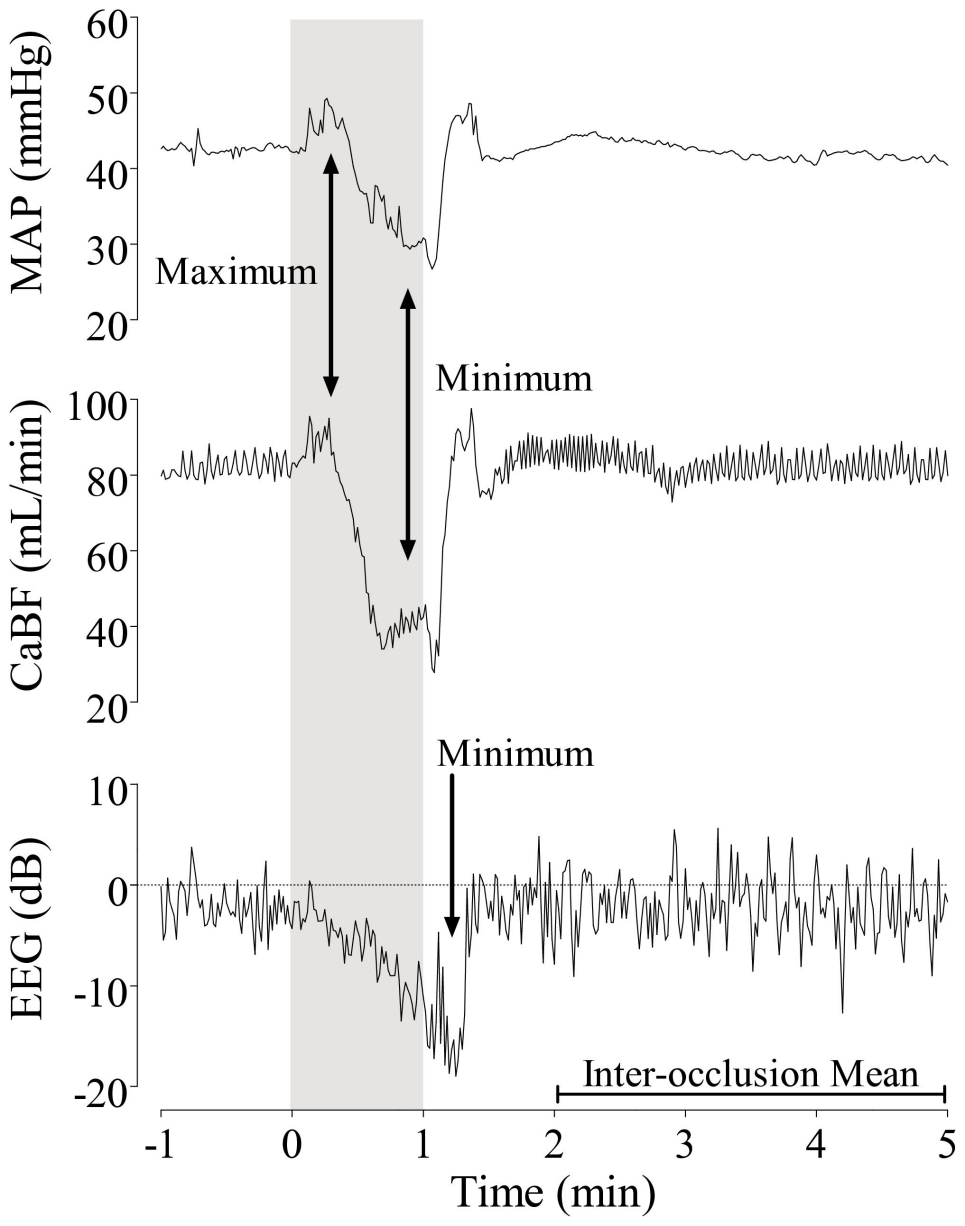
Time sequence of one second changes in mean arterial pressure (MAP, mmHg, top panel), carotid blood flow (CaBF, mL/min, middle panel), and EEG activity (EEG, dB, bottom panel) during one occlusion from the fourth quarter. The grey area indicates the one minute occlusion. Figure 1A represents a healthy normoxic fetus, responding with initial hypertension and cephalic hyperperfusion, which both progressively resolve with ongoing occlusion. Note the gradual suppression of EEG activity, reaching a nadir immediately after release of the occlusion, followed by rapid recovery to baseline values. Figure 1B represents a severely compromised fetus with pre-existing hypoxia, which also responds to occlusion with immediate, but short-lasting, hypertension and cephalic hyperperfusion, followed by progressively severe systemic hypotension and cephalic hypoperfusion. In addition, there is greater suppression of EEG activity that is superimposed on an already suppressed EEG background. The maximum MAP and CaBF at the beginning of each occlusion, and the minimum MAP and CaBF at the end of each occlusion were determined, as indicated by arrows. The nadir of EEG activity during or within 30 seconds after release of occlusion was determined, and the mean inter-occlusion values from the 2^nd^ to the 5^th^ minute were calculated for MAP, CaBF and EEG.

**Figure 2:**
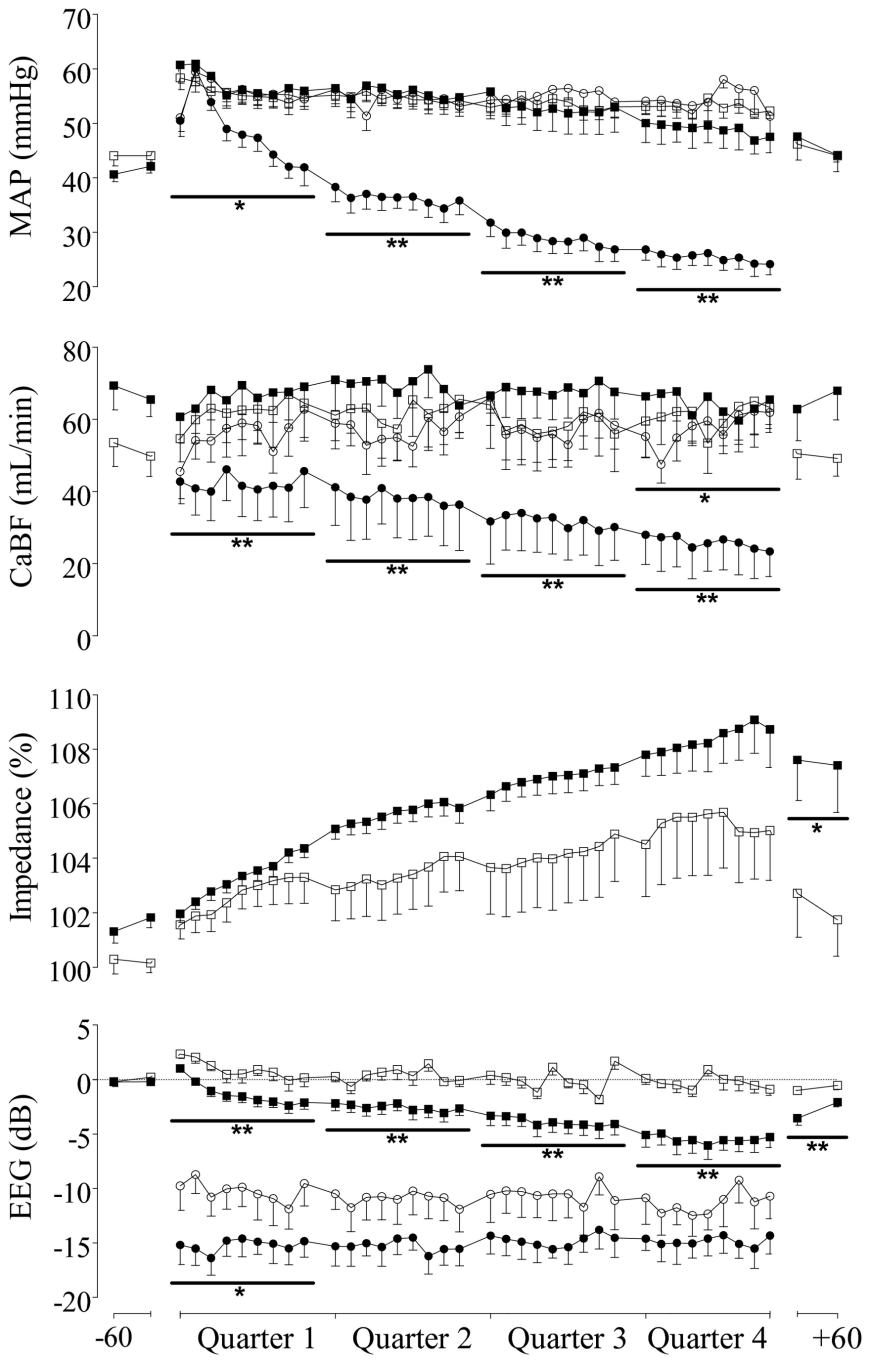
Changes in mean arterial pressure (MAP, mmHg, top panel), carotid blood flow (CaBF, mL/min, upper middle panel), impedance (Impedance, % baseline, lower middle panel), and EEG power (EEG, dB, bottom panel) in normoxic (open symbols) and hypoxic (closed symbols) fetuses exposed to one minute occlusions of the umbilical cord repeated every five minutes for four hours. MAP and CaBF are presented as the minimum during each occlusion (circles), and mean between occlusions (squares). Impedance is presented as the maximum (squares) during each occlusion. EEG activity is presented as the minimum (circles) during each occlusion, and mean (squares) between occlusions. The occlusion series is presented in four quarters (of nine occlusions each), with the baseline and recovery period presented in 30 min averages. Data are Mean ± SEM. *p<0.05, **p<0.01 vs. normoxic group.

Brief repeated umbilical cord occlusions were associated with a consistent systemic response marked by an initial rise in MAP, followed by a gradual fall as occlusion continued (Figure 1). In both groups, the maximum MAP during occlusions rose above baseline values from the first occlusion onwards (p<0.05), with no further change over time between the first and final quarter (normoxic group: 67.9 ± 6.8 vs. 67.4 ± 5.1 mmHg, hypoxic group: 64.5 ± 7.3 vs. 58.5 ± 13.7 mmHg; N.S.). In the normoxic group minimum MAP during occlusions also rose consistently above baseline values throughout the occlusion series (p<0.05), whereas, in the hypoxic group, minimum MAP only rose briefly above baseline values during the first quarter (p<0.05 vs. baseline and normoxic group, Figure 2); and then fell progressively below baseline with ongoing occlusions (p<0.05 vs. baseline for quarter 3 and 4, p<0.01 vs. normoxic group for all quarters), developing severe hypotension during the final quarter (24.1 ± 2.0 vs. 51.3 ± 3.7 mmHg; p<0.01 vs. normoxic group, last occlusion). Between occlusions, MAP was maintained above baseline values (p<0.05), with no difference between groups (N.S.).

Brief repeated cord occlusions were associated with an initial rapid increase in CaBF, followed by a gradual fall in blood flow as the occlusion continued (Figure 1). During occlusions, the maximum CaBF immediately rose above baseline values in both groups (p<0.05), and was significantly higher in the hypoxic group (p<0.05; quarter 2, p<0.01; quarter 1, 3, and 4). There was no further change in maximum CaBF within either group between the first and final quarter (normoxic group: 83.5 ± 21.1 vs. 85.6 ± 22.6 mL/min, hypoxic group: 81.7 ± 23.2 vs. 75.8 ± 21.0 mL/min; N.S.). However, whereas the minimum CaBF during occlusions was maintained around baseline values in the normoxic group, the minimum CaBF in the hypoxic group fell well below baseline values from the first occlusion onwards (p<0.05), and continued to decline throughout the occlusion series (p<0.01 vs. normoxic group, Figure 2), ultimately developing severe cerebral hypoperfusion (23.4 ± 7.0 vs. 61.9 ± 5.5 mL/min; p<0.01 vs. normoxic group, last occlusion). Between occlusions, the CaBF rapidly recovered to baseline values in both groups, but was lower in the hypoxic group during the last quarter (p<0.05 vs. normoxic group). Cephalic oxygen delivery (CaBF x O_2_ct) was significantly lower in the hypoxic group at baseline and throughout the occlusion series than the normoxic group, and fell below baseline values after the start of occlusions (p<0.01, Table 2).

### EEG activity and Impedance during brief repeated occlusions

In both groups, the onset of each occlusion was associated with a rapid suppression of EEG activity (p<0.05 vs. baseline), reaching its nadir immediately after release of each occlusion (e.g. see Figure 1). The magnitude of this EEG suppression was greater in the hypoxic group during the first quarter (p<0.05), but not significantly different between groups thereafter (Figure 2). Between occlusions, EEG activity rapidly recovered to baseline values in all fetuses of the normoxic group for the entire duration of the occlusion series. In contrast, in the hypoxic group EEG activity between occlusions fell below baseline values (p<0.05), and became progressively more suppressed than in normoxic fetuses with ongoing occlusions (p<0.01, Figure 2). Peak impedance during umbilical cord occlusions gradually increased over time (p<0.05 for both groups vs. baseline for quarters 2, 3, and 4), with no significant overall difference between groups (N.S., Figure 2).

### MAP, CaBF, Impedance, and EEG activity during recovery from brief repeated occlusions.

**Figure 3:**
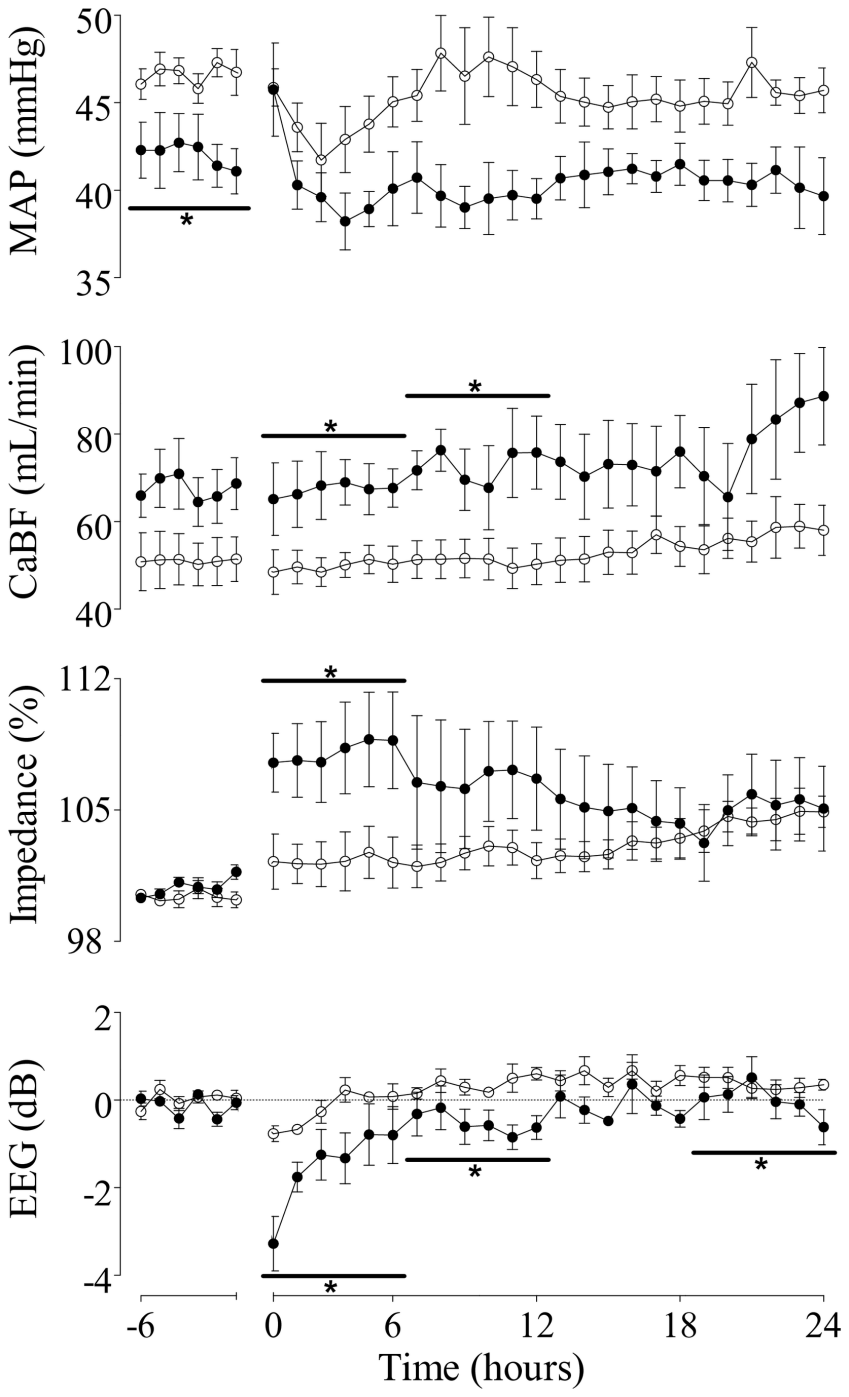
Time sequence of hourly changes in mean arterial pressure (MAP, mmHg, top panel), carotid blood flow (CaBF, mL/min, upper middle panel), impedance (Impedance, % baseline, lower middle panel), and EEG activity (EEG, dB, bottom panel) in normoxic (open circles) and hypoxic (closed circles) fetuses from 6 hours before, until 24 hours after a series of one minute occlusions of the umbilical cord repeated every five minutes (occlusion period omitted). Data are Mean ± SEM. *p<0.05 vs. normoxic group.

MAP was maintained at baseline values during the first 24 hours following occlusions, and was not significantly different between groups (N.S., Figure 3). Similarly, CaBF was also maintained around baseline values, but was significantly higher in the hypoxic group during the first 12 hours after the occlusion series (p<0.05 vs. normoxic group, Figure 3). Conversely, cephalic oxygen delivery during recovery remained significantly lower in the hypoxic group (p<0.01 vs. normoxic group, Table 2). Peak impedance was increased over baseline values in both groups for the 24 hour period of recovery (p<0.05), and was significantly higher in the hypoxic group during the first six hours of recovery (p<0.05 vs. normoxic group, Figure 3). Further, whereas EEG activity was maintained around baseline values in the normoxic group during the first 24 hours of recovery, there was an overall greater suppression of EEG activity in the hypoxic group during the first 12 hours, and again between 19 to 24 hours of recovery (p<0.05 vs. normoxic group, Figure 3).

### Seizures, Seizure-like events, and Sleep state cycling

Stereotypic seizures were seen in 2 of 8 fetuses in the hypoxic group, during the second and third quarter of occlusions, and in 4 of 8 fetuses during the final quarter of occlusions. Seizures continued in 3 of 8 fetuses during the first day, and 2 of 8 fetuses on the second day of recovery respectively (Table 3). On average seizures developed 147.5 ± 44.7 minutes after the occlusions started, with a seizure burden after the start of seizures of 26 ± 6 seconds per inter-occlusion period. After the occlusion series, the average seizure burden was 15.1 ± 3.4 minutes per hour over the first 6 hours of recovery. Subsequently fetuses showed a mean seizure burden of 15.3 ± 7.5 (from 7–12 hours), 6.8 ± 3.5 (from 13–18 hours), and 0.5 ± 0.5 (from 19–24 hours) minutes per hour. No seizures were observed in any fetus in the normoxic group. There was no significant difference in parameters during the baseline, last occlusion, or recovery period between fetuses that developed seizures and those that did not in the hypoxic group, although MAP, CaBF, and pH tended to be lower, while base deficit, lactate, impedance, and recovery of sleep state cycling tended to be higher in fetuses with seizures (N.S.). Occlusions were discontinued early (at occlusion 34, 39, and 45 respectively) in three fetuses with seizures.

**Table 3:** Seizure and seizure-like events in the Normoxic (N; n = 7) and Hypoxic (H; n = 8) groups.

		Baseline	Quarter 1	Quarter 2	Quarter 3	Quarter 4	Day 1	Day 2	Day 3	Total
Seizure Events	**N**	0	0	0	0	0	0	0	0	0
	**H**	0	0	10 (2)	9 (2)	16 (4)*	221 (3)	3 (2)	0	260 (4)*
Seizure	**N**	-	-	-	-	-	-	-	-	-
Duration (sec)	**H**	-	-	12.9±2.1	17.3±2.3	72.8±40.8	174.5±38.9	83.8±33.8	-	83.3±21.3
Seizure	**N**	-	-	-	-	-	-	-	-	-
Amplitude (µV)	**H**	-	-	152.9±22.5	173.5±14.3	257.4±47.6	153.1±34.1	110.6±30.5	-	168.1±8.9
Seizure-like	**N**	0	0	0	0	0	16 (2)	19 (3)	13 (3)	48 (3)
Events	**H**	15 (6)†	26 (6)†	27 (4)*	32 (5)*	44 (6)†	67 (6)	2 (1)	0	244 (8)*
Seizure-like	**N**	-	-	-	-	-	53.6±26.4	54.8±22.3	28.6±10.0	44.4±15.8
Duration (sec)	**H**	121.1±25.8	13.1±1.2	16.7±1.5	18.9±4.0	15.5±2.3	134.8±54.7	805±0.0	-	94.8±35.3
Seizure-like	**N**	-	-	-	-	-	70.8±22.2	82.5±18.9	66.3±15.1	75.0±11.8
Amplitude (µV)	**H**	54.9±4.7	133.9±29.9	125.2±39.3	126.3±16.5	129.1±29.2	70.5±7.8	25.0±0.0	-	96.5±14.0

Seizure and seizure-like events are presented as number of events per time period (number of affected fetuses), and total events during experiment (including all occlusions). Duration and amplitude data are presented as mean ± SEM; between-group comparisons by Mann Whitney-U test. *P<0.05, ^†^P<0.01. Sec = seconds, µV = microvolt.

**Figure 4:**
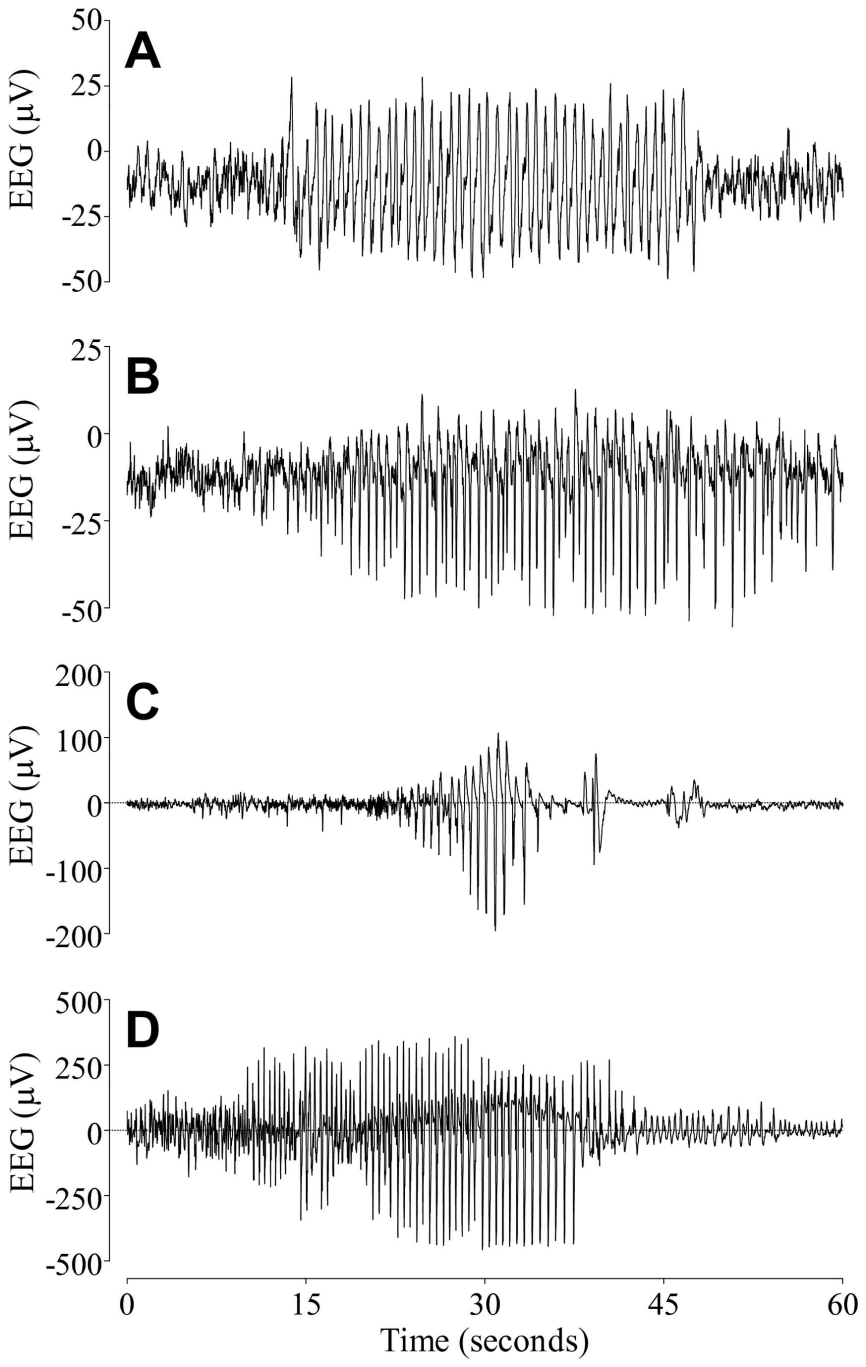
Examples of raw EEG data taken from individual fetuses with pre-existing hypoxia. (A) Seizure-like events in the baseline period. (B) Seizure-like events during recovery. (C) Overt seizures in the interocclusion period. (D) Overt seizures during recovery, after the occlusion series. Note the greater amplitude and the characteristic evolving pattern of the stereographic seizures compared with the lower and more uniform spike amplitude of the seizure-like events.

**Figure 5:**
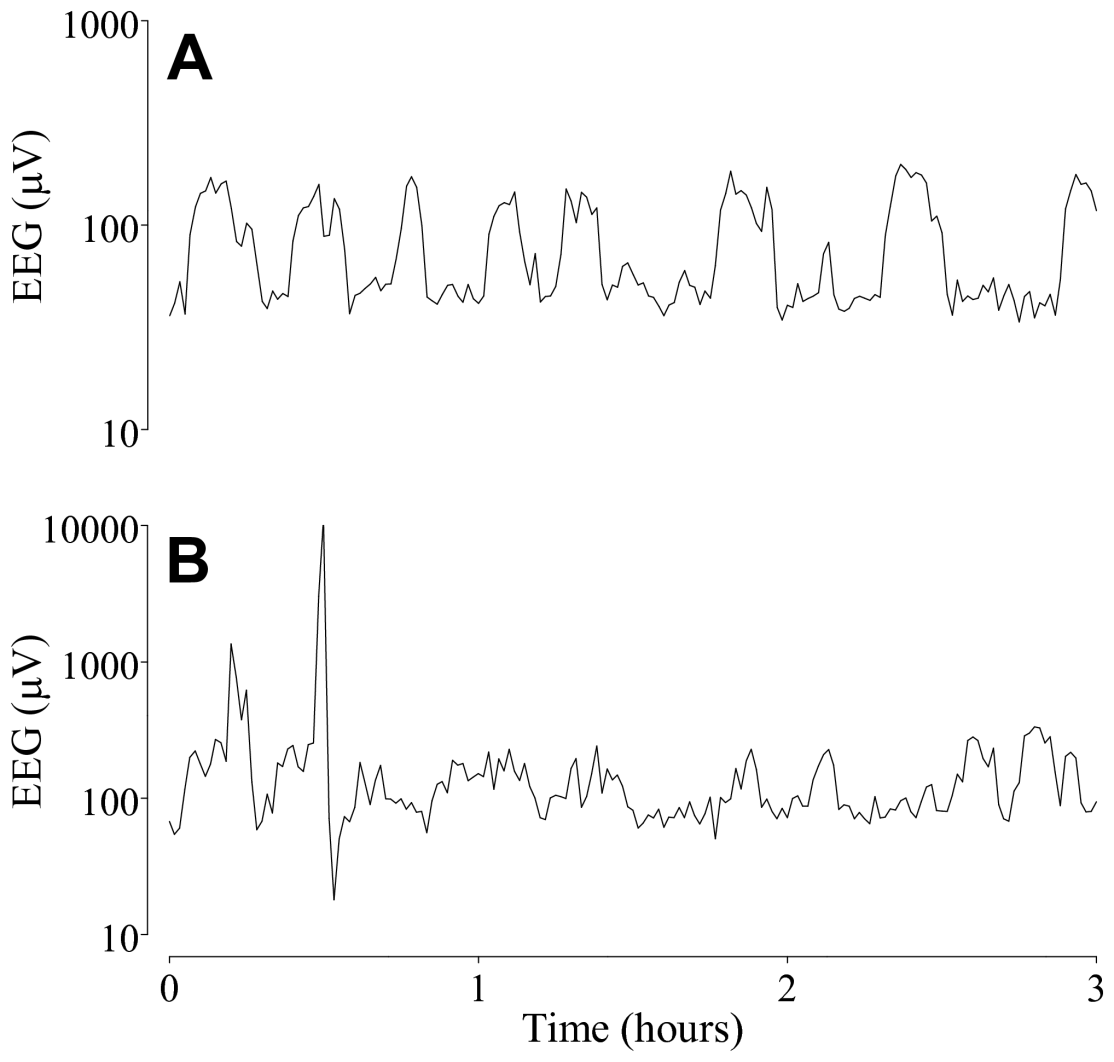
Examples of one minute EEG recordings from two fetuses. (A) Shows the alternating pattern of sleep state cycling that returned in this normoxic fetus within six hours after the occlusion series. (B) Shows absence of sleep state cycling 12 hours after the occlusion series in a fetus with pre-existing hypoxia.

**Figure 6:**
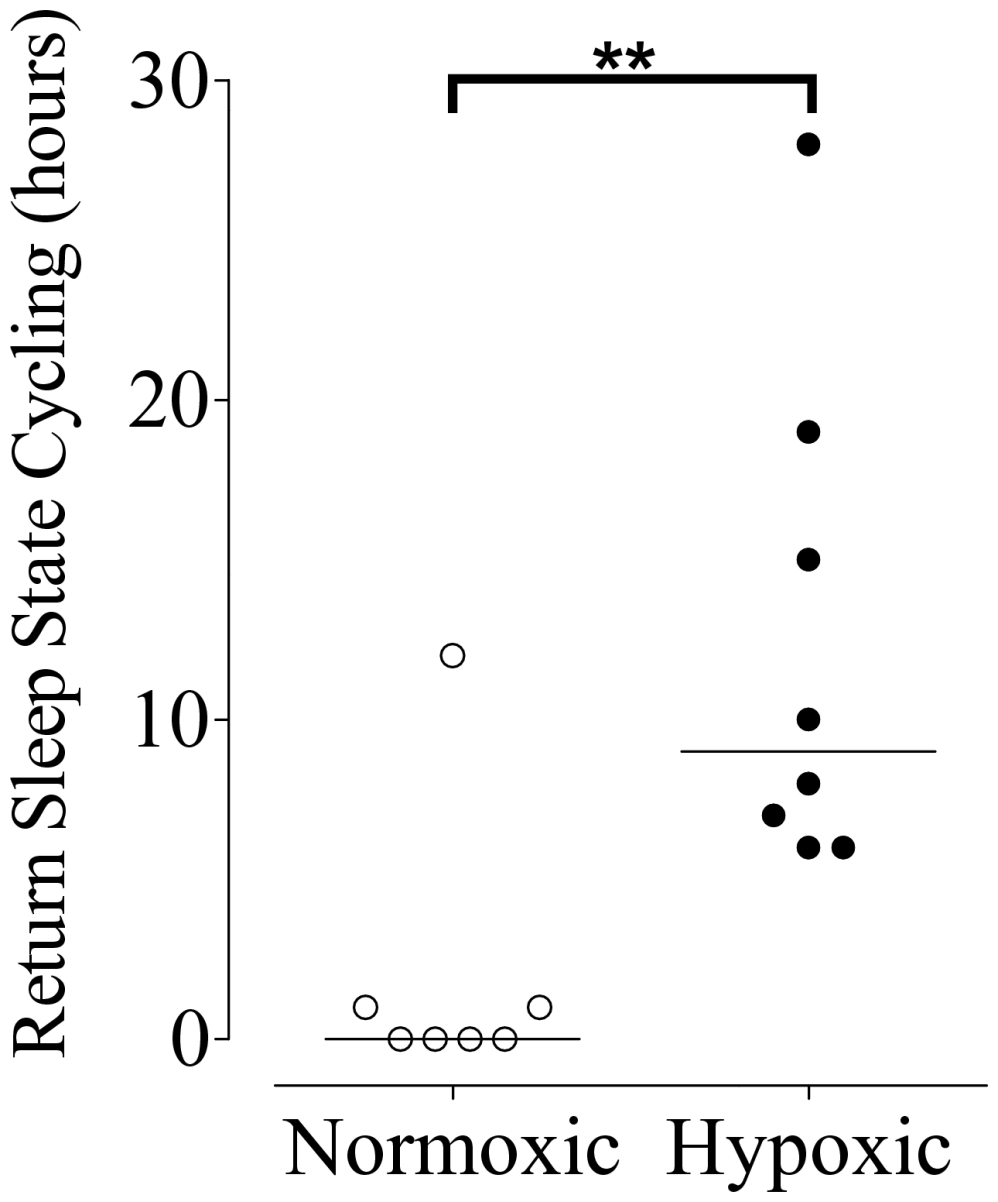
Scatter plot showing the time in hours that sleep state cycling returned normoxic (open symbols) and fetuses with spontaneous antenatal hypoxia (closed symbols) after the last occlusion. Horizontal bar indicates the median; **p<0.01 vs. normoxic group.

Seizure-like (epileptiform) events were seen in 8 of 8 fetuses in the hypoxic group during the baseline, occlusion period, and the first 2 days of recovery (Table 3). In contrast, seizure-like events were seen only in 3 of 7 fetuses in the normoxic group during the first 3 days of recovery, but not during the baseline or occlusion period. Examples of seizure-like events and stereotypic seizures are shown in Figure 4A, B, C, and D. Three fetuses in the normoxic group showed continued sleep state cycling despite occlusions. However, in all remaining fetuses, brief repeated occlusions were associated with rapid loss of sleep state cycling. After the occlusion series, sleep state cycling recovered in all but two animals from the hypoxic group (13 of 15 fetuses, Figure 5); recovery was significantly later in the hypoxic group (at a median of 9 vs. 0 hours after the final occlusion, p<0.01, Figure 6).

## Discussion

The present study demonstrates that compared with fetuses with normal blood gases before occlusions, fetal sheep with spontaneous antenatal hypoxia have evidence of severe neural compromise during brief repeated umbilical cord occlusions repeated at a rate consistent with early labor. Pre-existing fetal hypoxia was associated with development of severe metabolic acidosis, greater fall in arterial blood pressure and carotid blood during occlusions, with reduced cephalic oxygen delivery, profound EEG suppression during and between occlusions, and early-onset of evolving seizures between and after occlusions in a subset of fetuses.

The specific mechanisms of the progressive hypotension during repeated occlusions in fetuses with spontaneous antenatal hypoxia are likely to be multifactorial. Given that the ability of the fetus to survive prolonged asphyxia is closely linked to levels of cardiac glycogen [21], and fetal growth restriction is associated with reduced cardiac glycogen [22], it is likely that more rapid depletion of cardiac glycogen in the pre-existing hypoxia group was a major contributor to hypotension. Evolving myocardial injury and worsening fetal acidosis may have been additional factors, particularly in the later stages of the repeated occlusions [23].

In the present study, each (1-minute long) umbilical cord occlusion was associated with an initial, transient increase in carotid blood flow throughout the occlusion series in both groups. This is consistent with the well-recognized redistribution of blood flow to ‘central’ organs during hypoxia [24–27], and is known to be facilitated in part by nitric oxide [28]. In fetuses with pre-existing hypoxia this initial increase was followed by a marked fall in carotid blood flow well below baseline values even during the early period of hypertension, suggesting an actively mediated increase in carotid vascular resistance [29]. Previous studies have shown a comparable cerebrovascular response to umbilical cord occlusion and common uterine artery compression (flow reductions below 25% of baseline) in healthy near-term fetal sheep, with a moderate fall in carotid blood flow despite initial hypertension [29,30].

Perinatal neural injury after severe hypoxia/asphyxia is strongly associated with hypotension and impaired cerebral perfusion, as previously reviewed [5]. Consistent with this, in the present study, pre-existing hypoxia was associated with reduced cephalic oxygen delivery compared with the normoxic controls, related to markedly reduced blood oxygen content. In the baseline period, this was partially compensated for by increased carotid blood flow. During the occlusion series, oxygen delivery fell further in the pre-existing hypoxia group but not normoxic controls. The development of hypotension over time in the pre-existing hypoxia group was closely followed by a further parallel fall in carotid blood flow and cephalic oxygen delivery. This is consistent with earlier findings in near-term fetal lambs that the relationship between cerebral blood flow and arterial pressure is linear between approximately 18–45 mmHg, and that resting blood pressure is very close to the lower end of the autoregulatory range [31,32].

This impairment of oxygen delivery in fetuses with pre-existing hypoxia was associated with greater EEG suppression early during the occlusion series. The initial rapid-onset suppression of EEG activity during acute severe asphyxia or ischemia is a widely recognized, actively mediated, hypometabolic response in multiple settings, including fetal sheep [9,33], fetal llama [6], and adult rats [34], that helps delay neuronal depolarization and reduce neuronal injury [9]. This response has been attributed to increased extracellular release of multiple inhibitory neuromodulators including adenosine [9,34,35], noradrenergic alpha_2_-receptor activity [36], GABA [37], and inhibitory neurosteroids such as allopregnanolone [38,39]. Critically, our findings suggest that this immediate neuroprotective response continues during four hours of brief labor-like occlusions, even during severe systemic compromise in the spontaneously hypoxic fetal sheep. Our observations are consistent with previous studies in healthy fetal sheep that found a rapid profound but reversible suppression of EEG activity within 90 seconds from the start of each occlusion (4-minute occlusions repeated every 90 minutes for 6 hours, or 90 second occlusions repeated every 30 minutes for 3-5 hours), even though superior sagittal sinus blood flow or arterial blood pressure never fell below baseline values [10,11].

EEG activity between occlusions in our spontaneously hypoxic group became increasingly suppressed as occlusions continued. During prolonged umbilical cord occlusion the initial period of actively mediated EEG suppression, which can be reversed by blockade of the adenosine A1 receptor [9], is followed after several minutes by anoxic depolarization. Thus, it is plausible that in the present study, the increase in EEG suppression over time reflected developing anoxic depolarization. This is consistent with a previous report that pre-existing hypoxia in near-term sheep fetuses subjected to four 5-minute occlusions, 30 minutes apart, was associated with enhanced depression of electrocortical activity during and after occlusions, and greater striatal neural injury [40]. Supporting the concept of evolving neuronal depolarization, in the present study both groups showed a progressive, albeit small, increase in cytotoxic edema, as measured by cortical impedance, that tended to be greater in the pre-existing hypoxia group.

Alternatively, it is possible that pre-existing hypoxia may have been associated with a greater increase in inhibitory neuromodulators during repeated umbilical cord occlusions. Extracellular adenosine levels are substantially up regulated in response to even infrequent repeated umbilical cord occlusions [35], and basal plasma adenosine concentrations are increased in the small-for-gestational-age human fetus, in proportion to lower umbilical venous pO_2_ and pH [41]. Adenosine is quickly broken down by adenosine deaminase and the hypoxia induced release of adenosine rapidly returns to baseline during post-insult reoxygenation [42]. Nevertheless, other potent inhibitory neuromodulators such as GABA and allopregnanolone remain elevated for hours after cerebral ischemia or occlusion [38,43].

Stereotypic seizures were observed in the present study between occlusions and during the recovery period in four fetuses with pre-existing hypoxia, but never in the normoxic group. Shorter, seizure-like events were also seen in the pre-hypoxic group during and after the occlusion series, but only during the recovery period in the normoxic group. The fetuses with early onset seizures appeared to have somewhat worse blood composition and physiological parameters, however, this needs to be confirmed in a larger cohort. Although the pathological significance of seizure-like events remains unclear at present, clinically, the presence of seizures in term neonates with hypoxic ischemic encephalopathy is highly associated with poor neurological outcome [44,45]. Further, the spontaneously hypoxic group developed prolonged suppression of EEG activity for up to 12 hours after the occlusions, with delayed recovery of cortical impedance for at least 6 hours, suggesting that cellular membrane function was more severely compromised. Suppression of EEG amplitude and delayed recovery of sleep wake cycling in neonates with mild to moderate hypoxic-ischemic encephalopathy are strongly associated with adverse outcome [46].

A limitation of the current study is that histology could not be performed because of the high mortality in the group with pre-existing hypoxia. Previous studies have demonstrated mild to moderate selective neural injury from repeated occlusions in healthy near-term fetal sheep [13,47], albeit after more prolonged or more frequent episodes of repeated asphyxia than in the present study. Both the duration to EEG recovery and number of seizures were related to severity of neuronal loss [47]. Further, de Haan et al found that fetuses that developed cortical focal infarction from brief repeated occlusions exhibited more aberrant EEG activity [13], and required a much longer period to return to sleep state cycling than fetuses with only selective neural loss, even though cortical impedance was not significantly different. This is in agreement with our findings of early onset of seizures and delayed recovery of sleep state cycling in fetuses with pre-existing hypoxia compared with normoxic fetuses.

This study examined the effects of spontaneous antenatal hypoxia in near-term fetal sheep, similarly to Gardner et al. [48]. The limitation of this approach is that it is not possible to determine precisely when fetal hypoxia began. Potentially, for example, it could be a consequence of surgery, although no complications were evident at post-mortem. The pre-existing hypoxia group were lighter, even excluding the fetus from a triplet pregnancy, and had a mild but significant basal acidosis with hypercarbia and elevated lactate levels consistent with significant chronic placental impairment, as reported in clinical studies [1,49,50]. Although nearly all fetuses with pre-existing hypoxia were from multiple pregnancies (7/8), this was not significantly different from the normoxic group (4/8). This combination of findings in our hypoxic group suggests longstanding growth restriction related to chronic ‘physiological’ placental dysfunction, consistent with strong clinical evidence that growth restriction is associated with both chronic antenatal hypoxia and a higher risk of death and abnormal neurodevelopmental outcomes [1,2].

It is interesting to contrast the present study with previous reports that exposure to non-injurious hypoxia or ischemia can protect (“pre-condition”) against subsequent more severe insults [51]. This likely reflects two key differences. First, protection associated with preconditioning is highly time dependent. Typically protection is maximal after 3 to 7 days, and then attenuates [51]. Thus, in the context of long-standing antenatal hypoxia we would predict little or no neural protection. Second, in the current study, spontaneous hypoxia was ongoing, and associated with cerebrovascular compromise during the subsequent period of repeated brief asphyxia. This is in contrast with the typical strategy of brief exposure to the conditioning period of hypoxia well before the main insult, in studies of pre-conditioning. Thus, it may be that even if there was a putative ‘protective’ effect in the present study, it may have been masked by greater severity of cardiovascular compromise.

In summary, the present study shows that pre-existing hypoxia leads to exacerbation of cephalic hypoperfusion during brief repeated umbilical cord occlusions, with increased suppression of EEG activity between occlusions in near-term fetal sheep. The rapid suppression of EEG activity during each occlusion remained intact despite pre-existing hypoxia, however, the early onset of seizures and seizure-like events between occlusions in a subset of fetuses, and reduced recovery of EEG activity and cortical edema after the end of occlusions, strongly indicates greater vulnerability to neural injury during early labor-like asphyxia in fetuses with spontaneous, pre-existing hypoxia.
